# Eu, Gd-Codoped Yttria Nanoprobes for Optical and T_1_-Weighted Magnetic Resonance Imaging

**DOI:** 10.3390/nano7020035

**Published:** 2017-02-10

**Authors:** Timur Sh Atabaev, Jong Ho Lee, Yong Cheol Shin, Dong-Wook Han, Ki Seok Choo, Ung Bae Jeon, Jae Yeon Hwang, Jeong A. Yeom, Hyung-Kook Kim, Yoon-Hwae Hwang

**Affiliations:** 1 Department of Physics and Astronomy, Seoul National University, Seoul 08826, Korea; 2 Center for Biomaterials, Biomedical Research Institute, Korea Institute of Science and Technology, Seoul 02792, Korea; pignunssob@naver.com; 3 Department of Cogno-Mechatronics Engineering, Pusan National University, Busan 46241, Korea; choel15@naver.com; 4 Department of Radiology, Pusan National University Yangsan Hospital, Yangsan 50612, Korea; kschoo0618@naver.com (K.S.C.); junwb73@hanmail.net (U.B.J.); yhwang79@gmail.com (J.Y.H.); sigmajeonga@hanmail.net (J.A.Y.); 5 Department of Nano Energy Engineering, Pusan National University, Miryang 50463, Korea

**Keywords:** yttria, nanoprobes, optical imaging, magnetic resonance imaging, cytotoxicity, T_1_-weighted contrast agent

## Abstract

Nanoprobes with multimodal functionality have attracted significant interest recently because of their potential applications in nanomedicine. This paper reports the successful development of lanthanide-doped Y_2_O_3_ nanoprobes for potential applications in optical and magnetic resonance (MR) imaging. The morphology, structural, and optical properties of these nanoprobes were characterized by transmission electron microscope (TEM), field emission scanning electron microscope (FESEM), X-ray diffraction (XRD), energy-dispersive X-ray (EDX), and photoluminescence (PL). The cytotoxicity test showed that the prepared lanthanide-doped Y_2_O_3_ nanoprobes have good biocompatibility. The obvious contrast enhancement in the T_1_-weighted MR images suggested that these nanoprobes can be used as a positive contrast agent in MRI. In addition, the clear fluorescence images of the L-929 cells incubated with the nanoprobes highlight their potential for optical imaging. Overall, these results suggest that prepared lanthanide-doped Y_2_O_3_ nanoprobes can be used for simultaneous optical and MR imaging.

## 1. Introduction

In recent years, nanoprobes with multimodal functionality have been investigated extensively for a range of biomedical applications, such as optical imaging (OI), magnetic resonance imaging (MRI), bioseparation, controlled drug delivery, etc. [1,2,3,4]. Among them, gadolinia nanoprobes are used widely to improve the T_1_-weighted contrast between different tissues [5,6,7,8]. These gadolinia nanoprobes can efficiently induce the longitudinal relaxation of water protons and brighten the imaging place. On the other hand, the main limitation for industrial applications of gadolinia nanoprobes is the high production cost of gadolinium due mainly to its rarity on Earth. To resolve this limitation, gadolinium ions can be doped into another earth-abundant and low-cost host matrix. Surface-localized gadolinium ions in the low-cost host matrix can also change the longitudinal relaxation of the nearby water protons for MRI contrast enhancement. The similarity in chemical properties make the matrix of yttrium oxide Y_2_O_3_ (yttria) a promising host candidate for lanthanide doping [9,10]. For example, recent studies have suggested that a homogeneous yttria-lanthanides solid mixture can be obtained using the homogeneous urea precipitation method [11,12]. In addition, yttria-based nanostructures can potentially be used for optical biomaging purposes [13,14].

To the best of the authors’ knowledge, Eu^3+^ and Gd^3+^ codoped yttrium oxide nanospheres were not utilized as potential nanoprobes for simultaneous optical and magnetic resonance imaging. Therefore, the main aim of this study was to explore the possibilities of lanthanide-doped Y_2_O_3_ nanoprobes for bimodal imaging. The optical properties and MR relaxivity rate of the prepared lanthanide-doped Y_2_O_3_ nanoprobes were examined as a function of the Gd^3+^ concentration. The results are expected to make a strong contribution to other nanoprobes’ development.

## 2. Results and Discussion

**Figure 1:**
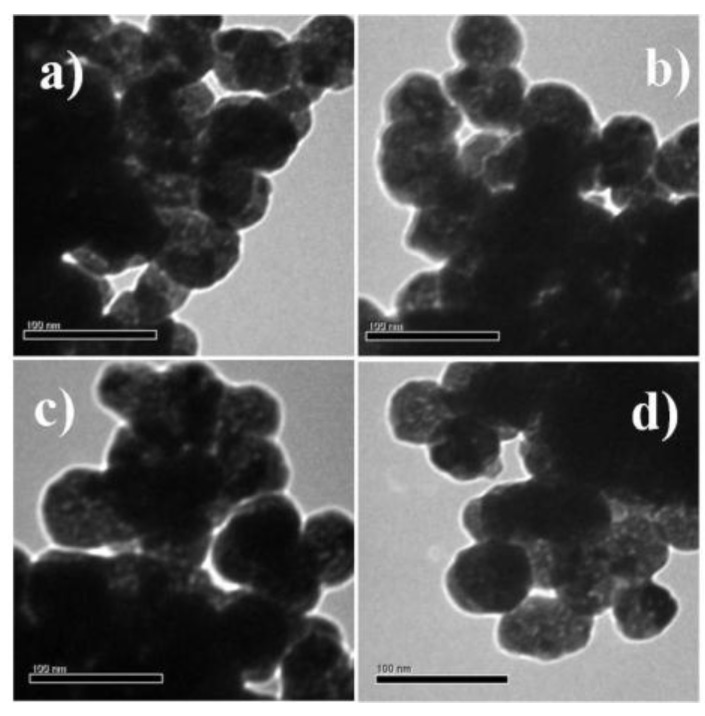
Transmission electron microscope (TEM) images of (**a**) bare Y_2_O_3_:Eu^3+^; (**b**) 3 mol % Gd^3+^ codoped Y_2_O_3_:Eu^3+^; (**c**) 7 mol % Gd^3+^ codoped Y_2_O_3_:Eu^3+^; and (**d**) 10 mol % Gd^3+^ codoped Y_2_O_3_:Eu^3+^.

For a comparative study of the optical and magnetic properties, samples with different Gd^3+^ codoping concentrations (0, 3, 7 and 10 mol %) were prepared. Transmission electron microscope (TEM), field emission scanning electron microscope (FESEM) and energy-dispersive X-ray (EDX) were used to examine the morphology and chemical composition of the samples. Figure 1a–d shows that all nanoprobes prepared had a spherical morphology within the range, 61–69 nm. For example, the mean diameters of the 10 mol % Gd^3+^ codoped nanoprobes, as measured by dynamic light scattering (DLS) (Figure S1, Supplementary Materials) were in accordance with the estimated sizes from FESEM analysis (Figure S2, Supplementary Materials). Obviously, codoping with Gd^3+^ did not alter the morphology, even at relatively high Gd^3+^ codoping concentrations as shown by FESEM measurements (Figure S2, Supplementary Materials). Bare Y_2_O_3_:Eu^3+^ and 3 mol % Gd^3+^ codoped Y_2_O_3_:Eu^3+^ nanoparticles were used to determine the elemental composition. EDX (Figure S3, Supplementary Materials) clearly indicated the presence of specific dopants (Eu in Y_2_O_3_:Eu^3+^) and (Eu, Gd in Y_2_O_3_:Eu^3+^, Gd^3+^) in the synthesized nanoparticles. Zeta potential was further measured at pH 7.5 to check the colloidal stability of the prepared samples. Measurement results show that zeta potential for all nanoprobes were in the range of 7–9 mV. Thus, prepared colloidal solutions of nanoprobes were stable for several hours only. However, homogeneous suspensions were formed again when solutions were ultrasonicated for several seconds. It is worth to mention that special surface coating can increase their colloidal stability in biological environment [6,8]. However, one should keep in mind that surface coating may affect the T_1_-relaxivity of these nanoprobes. 

Figure S4 (Supplementary Materials) shows typical XRD patterns of the prepared samples. The X-ray diffraction (XRD) peaks for bare Y_2_O_3_:Eu^3+^ were assigned to the standard cubic structure of Y_2_O_3_ (JCPDS No. 86-1107, space group *Ia3* (206)). A careful examination of the Gd-codoped samples showed that the position of all the XRD peaks shifted slightly towards lower angles. On the other hand, the samples still retained the cubic structure of Y_2_O_3_. The observed similarity with the Y_2_O_3_ structure suggests that the Gd-codoped samples are solid Y_2_O_3_-based solutions rather than mechanical mixtures of Y_2_O_3_, Eu_2_O_3_, and Gd_2_O_3_.

**Figure 2:**
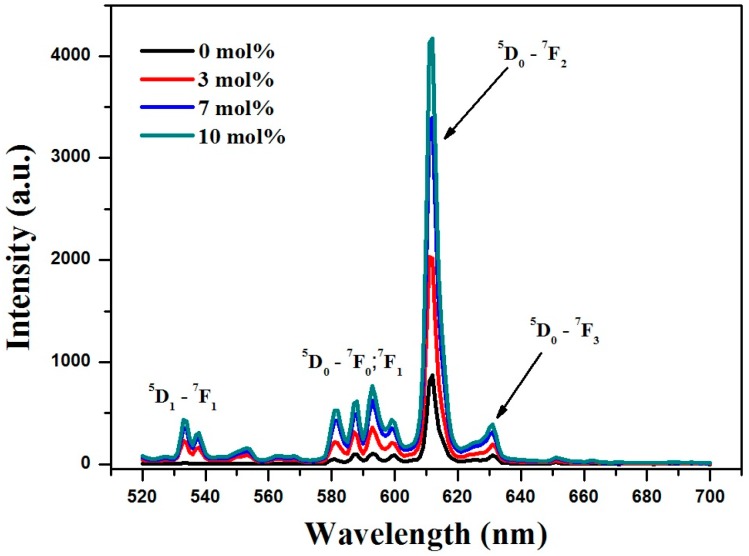
Photoluminescence (PL) emission spectra of prepared samples.

The prepared samples were tested further by photoluminescence (PL) at room temperature. Although the main reason for Gd-codoping was to achieve the paramagnetic functionality of bare Y_2_O_3_:Eu^3+^, Gd-codoping had some interesting effects on PL emission. All samples were measured under identical conditions so that the emission ratio could be compared. Figure 2 shows the PL emission spectrum revealed several main groups of emission lines, which were assigned to the ^5^D_1_ → ^7^F_1_ and ^5^D_0_ → ^7^F*_j_* (where *j* = 0, 1, 2, 3) transitions within the Eu^3+^ [12,13,14]. Partial replacement of Y^3+^ with Gd^3+^ in the host matrix allows easier charge transfer from Gd^3+^ to Eu^3+^ [15]. Therefore, Gd-codoping does not alter the peak position, but greatly improves the PL emission intensity. For example, the relative intensity of the strongest ^5^D_0_ → ^7^F_2_ (at 612 nm) peak increased monotonically with increasing Gd-concentration, which is in good agreement with the previously reported study [12].

**Figure 3:**
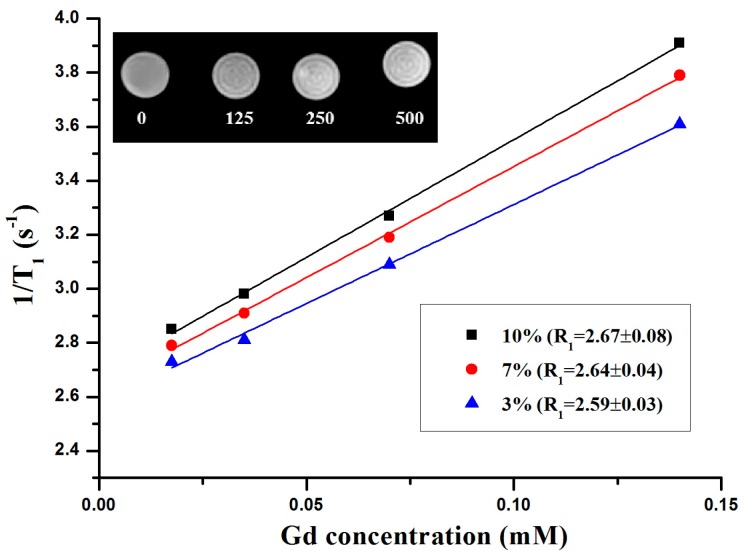
Longitudinal relaxivity rate R_1_ vs. various concentrations of Gd-codoped nanoparticles measured at room-temperature. Inset is T_1_-weighted images of the 10 mol % Gd^3+^ codoped nanoparticles at various concentrations (ppm).

A 1.5 T clinical MRI scanner was used to demonstrate the applicability of the Gd-codoped samples for T_1_-weighted MR imaging. The slope of the linear fit of 1/T_1_ vs. the nanoparticle concentration yielded a longitudinal relaxivity (R_1_) of the samples. Figure 3 shows that the R_1_ values of the 3%, 7%, and 10% codoped samples were approximately 2.59 ± 0.03, 2.64 ± 0.04, and 2.67 ± 0.08 s^−1^·mM^−1^ respectively. As expected, the R_1_ value increased with increasing Gd-concentration in the samples. Figure 3 (inset) shows that the T_1_-relaxation time of the water protons was reduced significantly, and the T_1_-weighted images became brighter with increasing concentration of 10 mol % Gd^3+^ codoped nanoparticles. The resulting R_1_ values were comparable to commercially available Gd chelates, such as gadopentetic acid Gd-DTPA (~3 s^−1^·mM^−1^) [16], which means that these nanoparticles can be used as bimodal contrast agents for MR and optical imaging.

**Figure 4:**
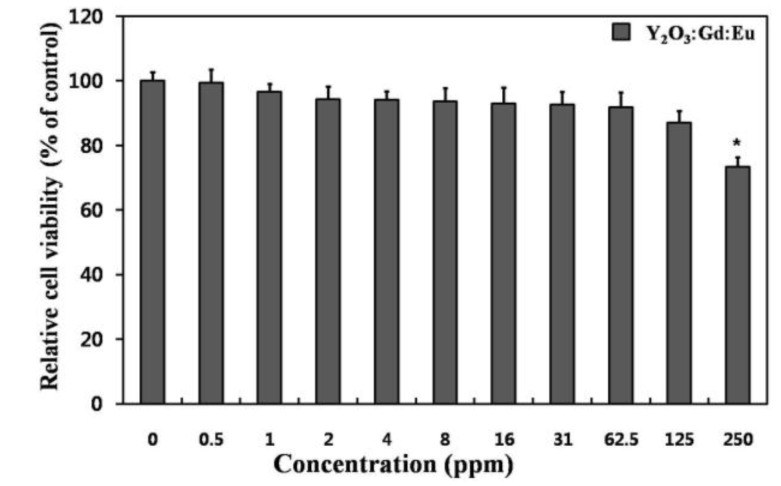
Relative cell viability of L-929 cells exposed to increasing concentrations (0–250 ppm) of the 10 mol % Gd^3+^ codoped nanoparticles. An asterisk (*) denotes a significant difference compared with the control, *p* < 0.05.

The cytotoxicity was measured to test the biosafety of the 10 mol % Gd^3+^ codoped nanoparticles. Figure 4 shows the cytotoxicity profiles of the nanoparticles in L-929 fibroblastic cells, which were determined using a WST-8 assay. The L-929 fibroblastic cells showed a noticeable concentration-dependent decrease in their relative cell viability. The prepared nanoparticles caused no significant decrease in cell viability at concentrations less than 60 ppm. This value is much higher than reported 8–10 ppm for Gd_2_O_3_-based nanoparticles [6,8]. Therefore, considering the in vitro cytotoxicity only, 10 mol % Gd^3+^ codoped nanoparticles can be used safely for bio-imaging at doses lower than 60 ppm.

**Figure 5:**
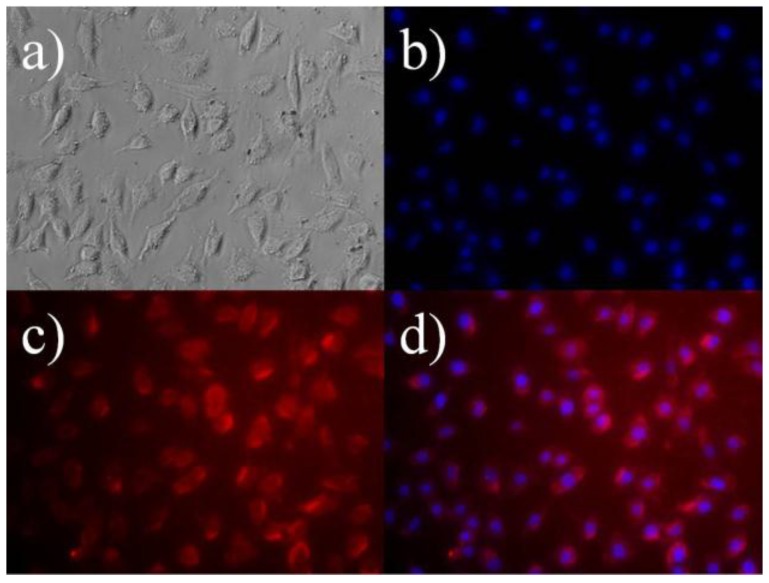
Fluorescence micrograps (200×) of L-929 cells treated with 10 ppm of 10 mol % Gd^3+^ codoped nanoparticles, followed by cell nuclei counterstaining with 10 μmol/L of 4’6-diamidino-2-phenylindole (DAPI). (**a**) Phase contrast image of the cells co-labelled with nanoparticles and DAPI; (**b**,**c**) Fluorescence images of the cells collected from DAPI (blue) and nanoprobes (red) respectively; (**d**) Merged image of (**b**,**c**).

To reveal the optical imaging potential of the 10 mol % Gd^3+^ codoped nanoparticles, a cultured monolayer of L-929 cells was incubated in the culture medium with a nanoparticle suspension at 10 ppm. Figure 5 shows that the L-929 cells grow with normal fibroblast-like morphologies after labeling with nanoparticles. The bright red fluorescence from the nanoparticles was observed mainly in the cytoplasm rather than inside the nuclei, suggesting that the nanoparticles could make cell imaging possible through efficient internalization into the cells with a uniform distribution in the cytoplasm. Therefore, the Gd-codoped nanoparticles can be utilized easily for clinical MRI applications and optical cell tracking.

## 3. Materials and Methods

### 3.1. Nanoprobes Preparation

Analytical graded Y_2_O_3_ (99.99%), Eu_2_O_3_ (99.99%), Gd_2_O_3_ (99.99%), HNO_3_ (70.0%), and urea (99.0%–100.5%) were purchased from Sigma-Aldrich (St. Louis, MO, USA) and used as received. Spherical Y_2_O_3_ nanoprobes co-doped with Eu^3+^ and Gd^3+^ were fabricated using a urea homogeneous precipitation method using the reported protocols [8,9,10,11,12]. Briefly, a sealed beaker with a freshly prepared aqueous solution of rare-earth nitrates (0.0005 mol in 40 mL of H_2_O) was placed into an electrical furnace and heated to 90 °C for 1.5 h. The dried synthesized precipitates were then calcined in air at 800 °C for 1 h to produce the oxide NPs. In all cases, the Eu^3+^ doping concentration was kept constant at 1 mol %, whereas the Gd^3+^ concentration was varied from 0 to 10 mol %.

### 3.2. Characterization

The structure of the prepared powders was examined by XRD Bruker D8 Discover (Billerica, MA, USA) using Cu-Kα radiation (λ = 0.15405 nm) at a 2θ scan range 20°–60° 2θ. The morphology of the particles was characterized by field emission scanning electron microscopy FESEM Carl Zeiss Supra 25 (Oberkochen, Germany) equipped with EDX. Size distribution and zeta potentials of the obtained nanoprobes were measured using a Nano ZS Zetasizer (Malvern, UK). The photoluminescence PL measurements were performed using a Hitachi F-7000 (Tokyo, Japan) spectrophotometer equipped with a 150 W Xenon lamp as the excitation source. The T_1_-weighted images were obtained using a 1.5 T MRI scanner Siemens (Munich, Germany) using the T_1_-weighted spin-echo method (TR/TE = 500 ms/15 ms, field of view (FOV) = 100 mm × 100 mm, slice thickness = 2 mm, matrix = 256 × 204, number of excitations (NEX) = 2). All measurements were performed at a room temperature of 22 ± 1 °C.

### 3.3. Cell Culture and Cytotoxicity Assay 

A murine fibroblast cell line (L-929 cells from subcutaneous connective tissue) was obtained from the American Type Culture Collection (ATCC CCL-1™, Rockville, MD, USA). The cells were routinely maintained in Dulbecco’s modified Eagle’s medium (Sigma-Aldrich, St. Louis, MO, USA), supplemented with 10% fetal bovine serum (Sigma-Aldrich, St. Louis, MO, USA) and 1% antibiotic antimycotic solution (including 10,000 units penicillin, 10 mg streptomycin and 25 mg amphotericin B per mL, Sigma-Aldrich, St. Louis, MO, USA) at 37 °C in 95% humidity and 5% CO_2_. The number of viable cells was indirectly quantified using highly water-soluble tetrazolium salt [WST-8,2-(2-methoxy-4-nitrophenyl)-3-(4-nitrophenyl)-5-(2,4-disulfophenyl)-2*H*-tetrazolium, monosodium salt] (Dojindo Lab., Kumamoto, Japan), reduced to a water-soluble formazan dye by mitochondrial dehydrogenases. The cell viability was found to be directly proportional to the metabolic reaction products obtained in WST-8. Briefly, the WST-8 assay was conducted as follows. L-929 cells were treated with increasing concentration (0–250 ppm) of nanoprobes and then incubated with WST-8 for the last 4 h of the culture periods (24 h) at 37 °C in the dark. Parallel sets of wells containing freshly cultured nontreated cells were regarded as negative controls. The absorbance was determined to be 450 nm using an ELISA reader (SpectraMax^®^ 340, Molecular Device Co., Sunnyvale, CA, USA). The relative cell viability was determined as the percentage ratio of the optical densities in the medium (containing the nanoprobes at each concentration) to that of the fresh control medium.

### 3.4. Fluorescence Microscopy

To examine the cellular uptake and distribution of nanoprobes within the L-929 cells and subsequent cell imaging, the cells were treated with 10 ppm of nanoprobes for 4 h. After treatment, the cells were fixed with 3.5% paraformaldehyde (Sigma-Aldrich) in 0.1 M phosphate buffer (pH = 7) for 10 min at room temperature and immediately observed under a fluorescence microscope (IX81-F72, Olympus Optical, Osaka, Japan).

### 3.5. Statistical Analysis

All variables were tested in three independent cultures for cytotoxicity assay, which was repeated twice (*n* = 6). Quantitative data are expressed as the mean ± standard deviation (SD). Data were tested for homogeneity of variances using the test of Levene, prior to statistical analysis. Statistical comparisons were carried out by a one-way analysis of variance (ANOVA), followed by a Bonferroni test for multiple comparisons. A value of *p* < 0.05 was considered statistically significant.

## 4. Conclusions 

In summary, Eu^3+^ and Gd^3+^ codoped Y_2_O_3_ nanoparticles were prepared for potential MRI and optical imaging applications. We showed that Gd-codoping into the Y_2_O_3_ host matrix resulted in PL emission enhancement. MRI relaxivity studies of the Gd-codoped samples suggested that the prepared nanoparticles can also be used for T_1_-weighted contrast enhancement. In addition, the cytotoxicity results showed that these nanoparticles are safe for bio-imaging at doses lower than 60 ppm. Therefore, the bimodal functionality and low toxicity makes these nanoparticles suitable for nanomedical applications.

## Supplementary Materials

The following are available online at http://www.mdpi.com/2079-4991/7/2/35/s1.
