# Supplementary material for: Eu, Gd-Codoped Yttria Nanoprobes for Optical and T1-Weighted Magnetic Resonance Imaging
Source: Nanomaterials (Basel). 2017 Feb 10;7(2):35. doi: 10.3390/nano7020035 (PMC5333020; doi:10.3390/nano7020035)
Supplement: Supplementary file 1 [file nanomaterials-07-00035-s001.pdf]

Supplementary Materials: Eu, Gd-Codoped Yttria Nanoprobes for Optical and T_1-Weighted Magnetic Resonance Imaging

Timur Sh Atabaev, Jong Ho Lee, Yong Cheol Shin, Dong-Wook Han, Ki Seok Choo, Ung Bae Jeon, Jae Yeon Hwang, Jeong A. Yeom, Hyung-Kook Kim and Yoon-Hwae Hwang

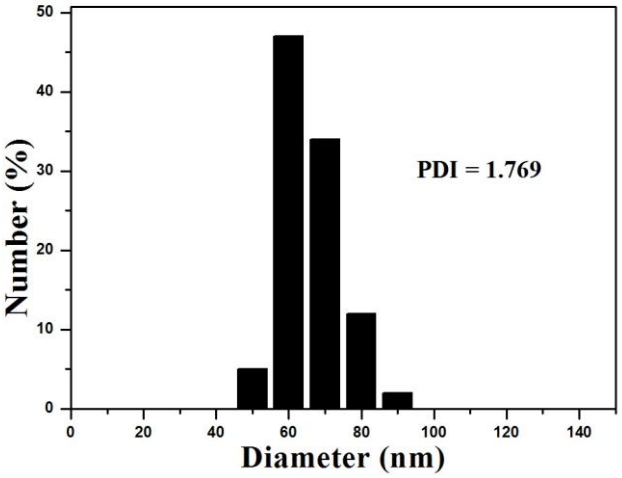

Figure S1. DLS measurements and PDI analysis of 10 mol % Gd^{3+} codoped $Y_2O_3:Eu^{3+}$.

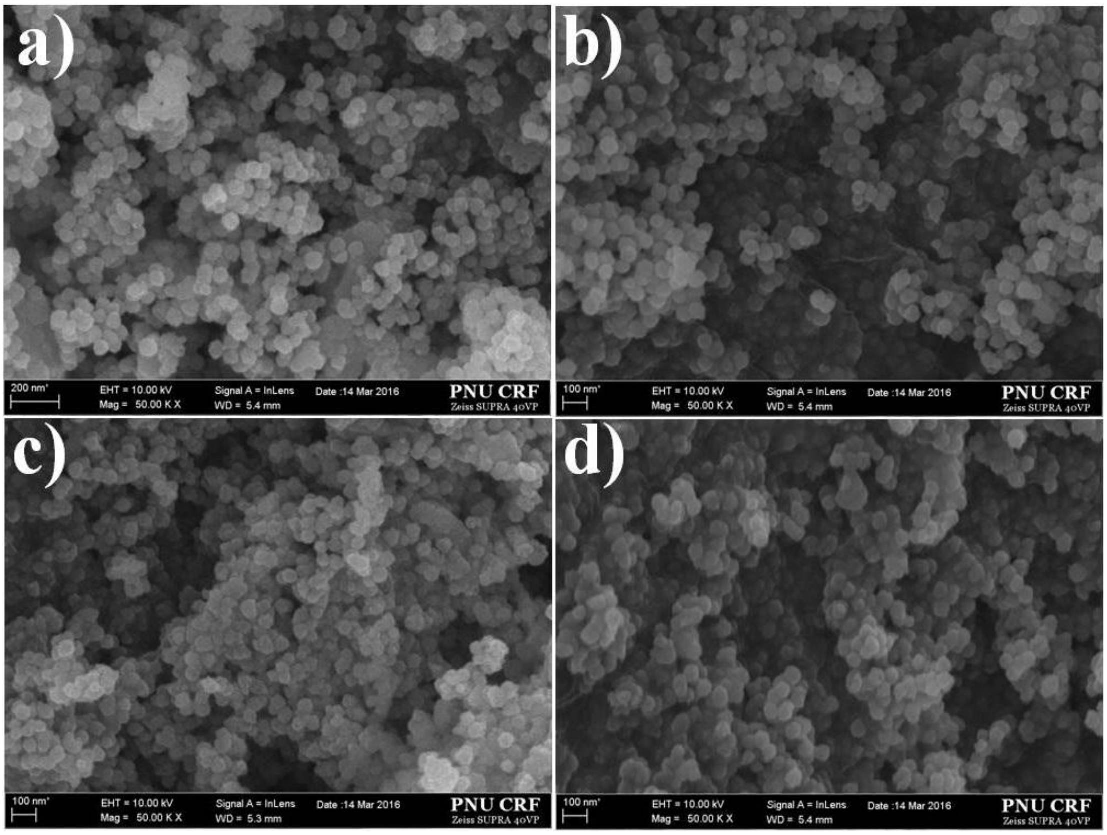

Figure S2. FESEM images of (**a**) bare $Y_2O_3:Eu^{3+}$; (**b**) 3 mol % Gd^{3+} codoped $Y_2O_3:Eu^{3+}$; (**c**) 7 mol % Gd^{3+} codoped $Y_2O_3:Eu^{3+}$; and (**d**) 10 mol % Gd^{3+} codoped $Y_2O_3:Eu^{3+}$.

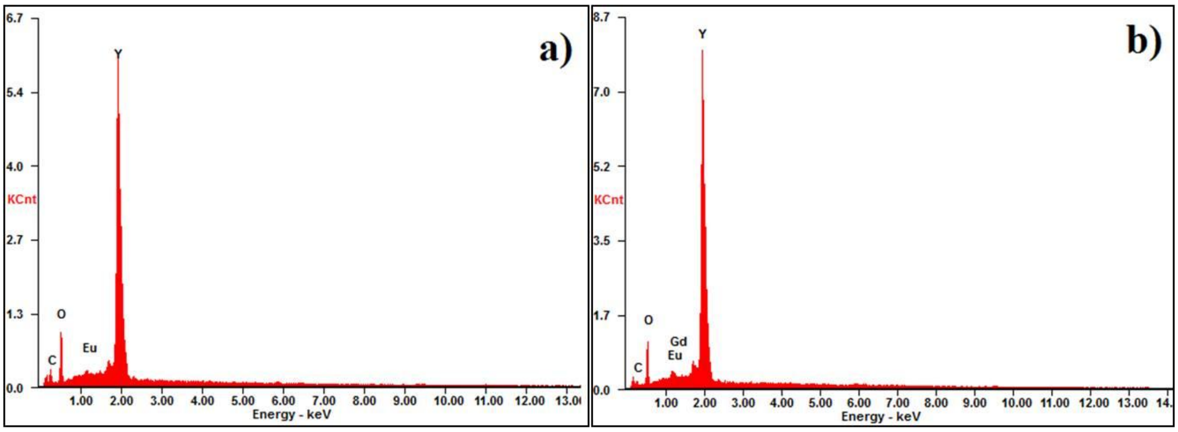

Figure S3. EDX analysis of (**a**) bare $Y_2O_3:Eu^{3+}$; and (**b**) 3 mol % Gd^{3+} codoped $Y_2O_3:Eu^{3+}$.

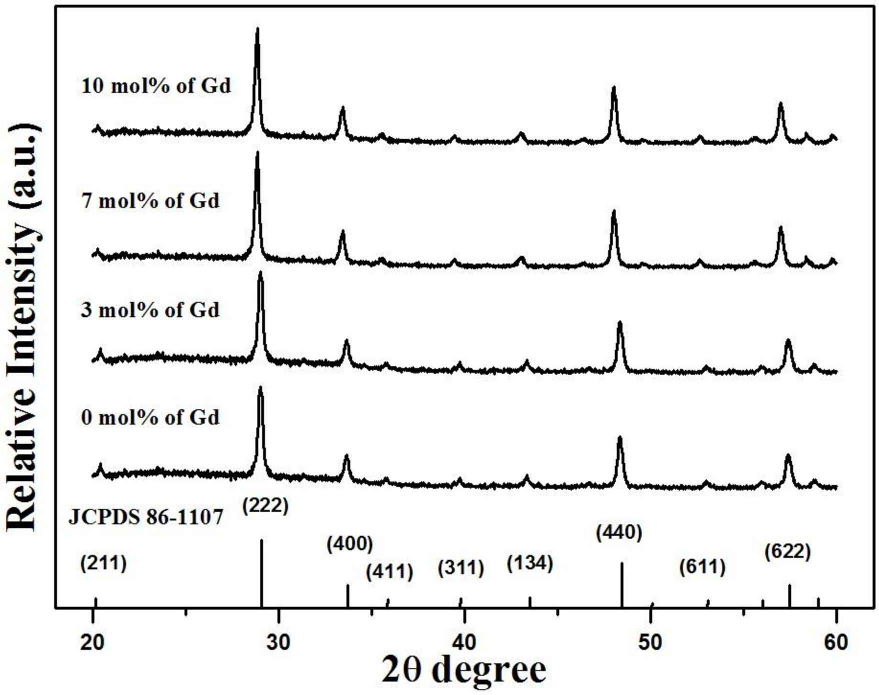

Figure S4. XRD patterns of prepared samples.
